# A Case of Non-cirrhotic Portal Hypertension in a Patient With Primary Myelofibrosis Disease

**DOI:** 10.7759/cureus.44313

**Published:** 2023-08-29

**Authors:** Ruchi Mangal, Maria Jamil, Zeinab Nasser, James P Purtell

**Affiliations:** 1 Internal Medicine, Henry Ford Health System, Detroit, USA

**Keywords:** portal vein thrombosis, low-molecular-weight heparin (lmwh), pelvic vein thrombosis, direct oral anticoagulants (doac), warfarin, therapeutic anticoagulation, myeloproliferative

## Abstract

Idiopathic non-cirrhotic portal hypertension can emerge due to a varied spectrum of underlying and contributory factors, presenting in the form of abdominal distention as the initial symptom encountered. Often, a patient remains asymptomatic to the underlying cause and seeks medical care for their abdominal enlargement. As the portal hypertension continues to progress, ascites begins to develop due to a history of portal vein thrombosis being sufficient to increase splanchnic blood flow in a portal hypertensive pattern. We present a rare case of ascites in a non-cirrhotic patient due to portal vein thrombus with underlying myeloproliferative disease of primary myelofibrosis.

## Introduction

Idiopathic non-cirrhotic portal hypertension can arise due to a multitude of underlying causes [1]. In some cases, it emerges secondary to the obliteration of portal venules and increased splanchnic blood flow in patients with immunological disorders, infections, toxin exposure, or thrombophilia [2]. Often, the first symptom patients experience in these cases is abdominal distention once the hypertension has progressed to ascites. The presence of massive ascites indicates a need for serologic workup revealing possible underlying malignancy and other conditions. Currently, the standard of treatment for non-cirrhotic portal hypertension is to treat the underlying cause [3]. Here, we present a rare case of ascites in a non-cirrhotic patient with underlying primary myelofibrotic disease.

## Case presentation

**Figure 1:**
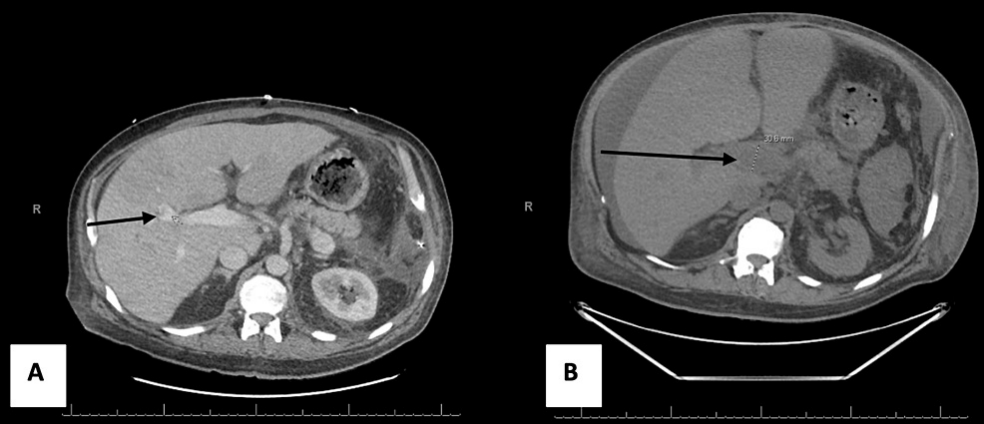
This figure compares two axial views of CT scans of the pulmonary abdomen pelvis taken in January 2023 (A) and April 2023 (B). In B, the thrombus within the branch of the right portal venous system clearly extends into the anterior segment. The progression of the portal vein thrombosis over the three-month period can be seen between A and B.

A 52-year-old male with a past medical history of Stage 3a chronic kidney disease, heart failure with a reduced ejection fraction of 46%, and primary myelofibrosis disorder presented for worsening abdominal distention in April 2023. The patient had begun noticing distention in his abdomen and presented to the emergency department, which found an extensive portal vein thrombus on ultrasound. The patient had a history of splenectomy in January 2023 due to progressive myelofibrosis. Computed tomography (CT) scans taken three months apart showed significant progression over these months to involvement of the entire portal and splenic veins (Figure 1).

**Figure 2:**
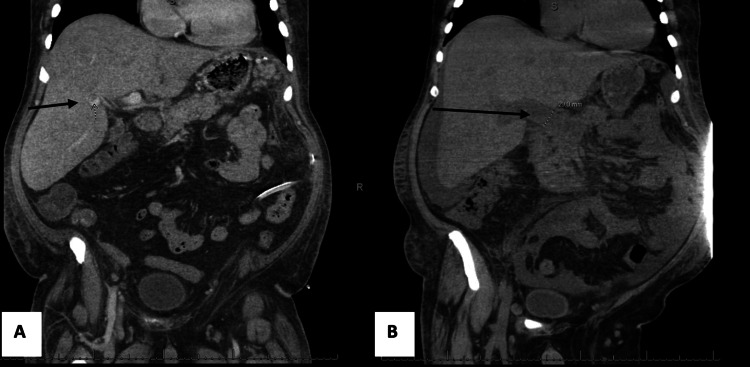
This figure compares two sagittal views of the CT scans of the pulmonary abdomen pelvis taken in January 2023 (A) and April 2023 (B). The images highlight the progression of the portal vein thrombosis over the three-month period. In (B), the entire portal vein and splenic vein are thrombosed with partial thrombosis of the superior mesenteric vein near the portal confluence.

He was diagnosed with primary myelofibrosis and osteosclerosis in December 2017 with grade 1-2/3 fibrosis with anemia, splenomegaly, and positive *JAK2 *mutation. Molecular studies showed TET2/CUX1/MTA3, which indicates a much more aggressive disease with higher chances of transformation. The patient underwent splenectomy in January 2023, and CT imaging three months post-surgery showed a thrombus within a branch of the right portal venous system extending into the anterior segment (Figure 2). He was initially started on high-intensity heparin after the surgery and discharged during that stay on Coumadin.

On presentation to the hospital three months post-splenectomy, as his international normalized ratio (INR) was supratherapeutic, high-intensity heparin was held and enoxaparin was started after the prothrombin time-INR decreased to below 3. Due to worsening distention, he underwent a paracentesis with the removal of 3.75 L of fluid. Fluid studies showed that the abdominal fluid was transudate in nature with a white blood cell count >250. Therefore, the patient was started on cefpodoxime 400 mg twice per day for spontaneous bacterial peritonitis for five days. He developed a worsening prerenal acute kidney injury and was started on albumin with all guideline-mediated medical therapy (GDMT) held. This treatment course caused the resolution of his symptoms. Due to symptom resolution, he was stable for discharge on enoxaparin, with his GDMT restarted. He was advised to follow up with his primary care provider to continue monitoring the progression of the abdominal distention and portal vein thrombus. Two months after this hospitalization, the patient had multiple falls while on enoxaparin, which led to a massive hemorrhage, and he ultimately passed away.

## Discussion

Idiopathic non-cirrhotic portal hypertension can be caused by a variety of liver diseases [1]. It can arise secondary to the obliteration of portal venules and increased splanchnic blood flow in patients with immunological disorders, infections, toxin exposure, or thrombophilia [2]. A history of portal vein thrombosis can be sufficient to increase splanchnic blood flow in a portal hypertensive pattern, eventually leading to ascites. Ascites can be the first sign of portal hypertension in those with normal liver makers due to preserved liver synthetic function [1].

Ascites is caused by cirrhotic portal hypertension in 80% of cases and by malignancy in approximately 10% of cases. Treating the underlying cause of non-cirrhotic portal hypertension is the mainstay of therapy currently. Patients can have massive ascites and spontaneous bacterial peritonitis with normal serologic workup allowing further investigation to identify the underlying cause, often malignancy [3]. Portal hypertension has been seen as a rare side effect of lymphomas or other myeloproliferative diseases, and it can be the first noticeable symptom to the patient in these cases. The mechanism behind this non-cirrhotic portal hypertension is that there is either an obstruction of portal venous return due to intrahepatic lymph stasis or portal vein thrombosis due to a hypercoagulable state [4].

Spontaneous bacterial peritonitis is a common complication of ascites, regardless of its cirrhotic or non-cirrhotic cause. While in cirrhotic patients, octreotide, midodrine, and albumin are necessary for treatment, in non-cirrhotic patients, there is still high ascitic fluid protein due to the liver production of albumin staying steady at baseline. Treatment of spontaneous bacterial peritonitis can be managed empirically with intravenous third-generation cephalosporin and albumin only [5].

Due to the liver’s synthetic function being preserved, identifying portal hypertension and monitoring its progression is a major challenge. Future studies should investigate possible screening guidelines for portal hypertension in patients with myeloproliferative disease. Additionally, further studies should be conducted on anticoagulation therapies that prevent these thrombi from forming in the splanchnic veins.

## Conclusions

Idiopathic non-cirrhotic portal hypertension has many distinct underlying causes. In this case, the patient had severe progression of their portal vein thrombus despite being on warfarin. The only noticeable symptom to the patient was abdominal distention, and the development of portal hypertension would have gone unnoticed without the apparent ascites. Myeloproliferative diseases and malignancy are prevalent causes of portal hypertension that often go unrecognized. The hypercoagulable state allows thrombus in the portal venules, leading to portal hypertension. Ascites is often the first symptom of portal hypertension in these cases, as seen in this case. Patients with these myeloproliferative conditions should be monitored for the development of this complication before the progression of ascites or spontaneous bacterial peritonitis.
